# The Safety of 12‐Weekly Monitoring of Neutrophil Count in Long‐Term Clozapine Patients

**DOI:** 10.1111/acps.13818

**Published:** 2025-05-03

**Authors:** David Taylor, Siobhan Gee, Marinka Helthuis, Ebenezer Oloyede

**Affiliations:** ^1^ Institute of Pharmaceutical Science King's College London London UK; ^2^ Pharmacy Department South London and Maudsley NHS Foundation Trust London UK; ^3^ Leyden Delta B.V Nijmegen the Netherlands

**Keywords:** agranulocytosis, blood monitoring, clozapine, neutropenia

## Abstract

**Introduction:**

Clozapine is the only truly effective treatment for refractory schizophrenia, but its use is constrained by the requirements for frequent monitoring of neutrophil counts. In the UK during the COVID‐19 pandemic, the frequency of clozapine blood monitoring was reduced in some units from 4‐weekly to 12‐weekly. We aimed to investigate the outcomes of reduced monitoring in long‐term clozapine patients.

**Methods:**

This was an anonymous, retrospective, observational cohort study. No restrictions were applied regarding care setting (i.e., outpatients or inpatients). All patients who registered for reduced frequency haematological monitoring from 1 March 2020 to 1 November 2022 were included and followed up till 1 August 2024. The primary outcome was death resulting from clozapine‐induced agranulocytosis (CIA). Secondary outcomes were the proportion of patients with mild to moderate neutropenia during the follow‐up period and the proportion of patients who reverted to standard monitoring during the study period.

**Results:**

Amongst 1025 patients, there were no cases of agranulocytosis over 3365.9 patient‐years of 12‐weekly blood monitoring (incident rate 0.0 per 100 person‐years). There were 43 episodes of mild neutropenia (so‐called amber results—1.5–2.0 × 10^9^/L) or neutropenia (red results < 1.5 × 10^9^/L), an overall incident rate of 1.28 per 100 person‐years. During follow‐up, 41 patients (4%) reverted permanently to standard 4‐weekly monitoring, and 157 patients (15%) temporarily interrupted reduced frequency monitoring but restarted 12‐weekly monitoring before the end of the follow‐up period. In total, 42 patients (4%) died during the observation period—no death was related to agranulocytosis.

**Conclusion:**

Reducing the frequency of clozapine haematological monitoring to 12‐weekly was safe in a group of long‐term patients. No cases of agranulocytosis occurred and no deaths due to agranulocytosis were recorded. Most patients remained on extended‐interval monitoring.

**Summary**

- Significant outcomes
  ○ This study demonstrated the safety of extending neutrophil monitoring frequency from monthly to 3‐monthly in patients who have been taking clozapine for at least 1 year.
  ○ Reducing haematological monitoring frequency did not increase the risk of missing cases of agranulocytosis.
  ○ Extending monitoring intervals could have a significant impact on persistence with clozapine treatment and, by extension, improve morbidity and mortality in this vulnerable patient group.
- Limitations
  ○ We did not include a control group of patients on standard monitoring.
  ○ Safety was demonstrated only over 3366 patient‐years.
  ○ Patients allowed to move to 3‐monthly monitoring were restricted to those with no prior neutropenic events.

## 1 Introduction

Clozapine is the only truly effective treatment for refractory schizophrenia, but its use is constrained by the requirements for frequent monitoring of neutrophil counts [1]. This monitoring is mandated in most countries because of the risk of agranulocytosis associated with clozapine use [2]. Although models differ somewhat, in most jurisdictions, monitoring is weekly during the first weeks of treatment before moving to biweekly or monthly monitoring in longer‐term treatment. For the most part, monitoring continues throughout the duration of clozapine use.

There are several aspects of clozapine blood monitoring that are subjects of controversy [3]. While it is unarguable that current monitoring schemes radically reduce the risk of death from agranulocytosis early in treatment, it is not clear that monthly monitoring in long‐term treatment is either necessary (given the low risk of agranulocytosis in these patients) or effective (clozapine‐induced agranulocytosis [CIA] is usually a rapidly developing event that is unlikely to be detected by infrequent monitoring) [4, 5]. It is also noteworthy that the mandated frequency of monitoring varies substantially across different countries, thus suggesting that not all schedules are evidence‐based or at least that they are not all based on the same evidence [2].

Standard UK monitoring frequencies of neutrophils are weekly counts for the first 18 weeks, then fortnightly for the remainder of the first year, and monthly thereafter. During the COVID pandemic, some healthcare units reduced the frequency of clozapine blood monitoring for patients who were on monthly monitoring (i.e., patients who had taken clozapine for a year or more) in an attempt to minimise the risk of transmission between healthcare workers and clozapine patients [6]. An early analysis of the safety of less frequent monitoring suggested that 12‐weekly monitoring might be sufficient to prevent fatal outcomes from clozapine‐induced blood dyscrasia [7]. Here we report on the safety of 12‐weekly monitoring in a larger cohort over a longer time period.

## 2 Aim

The aim of the study was to evaluate the safety of 12‐weekly blood monitoring in people taking long‐term clozapine.

## 3 Method

### 3.1 Data Source, Study Sample and Ethical Approval

This retrospective observational study was based on data from one of the three clozapine monitoring service databases in the United Kingdom (UK), Zaponex Treatment Access System (*ZTAS*) managed by *Leyden Delta BV*. Demographic and haematological data such as ethnicity, clozapine initiation date, and benign ethnic neutropenia (BEN) status were obtained from the ZTAS database, without access to patient identifiers to preserve anonymity. We included all patients registered for reduced monitoring from 1 March 2020 to 1 November 2022. We did not apply any restrictions regarding care setting (i.e., outpatients or inpatients). This was an anonymous, retrospective, observational database study. As such, patient consent was not required, and all procedures relating to this work comply with the ethical standards of the UK Health Research Authority (HRA). Strengthening the Reporting of Observational studies in Epidemiology (STROBE) reporting guidelines were followed (Data S1) [8].

### 3.2 Study Design

As agreed by expert consensus, eligibility criteria for reduced blood monitoring included no history of neutropenia (i.e., an absolute neutrophil count (ANC) ≥ 2.0 × 10^9^/L or ≥ 1.5 × 10^9^/L if there was a history of BEN) and adherence to clozapine prescription within the preceding year. Reduced frequency monitoring required completion of an off‐licence form as it was outside the terms of the Product Licence [9]. In this study, follow‐up started at the time of registering for reduced monitoring and ended either by discontinuation of treatment, loss to follow up (e.g., death) or the end of the study follow‐up (1 August 2024).

The primary outcome of interest was death resulting from CIA. The secondary outcome was the proportion of patients who developed haematological events during follow up. Haematological events were defined by Medicines and Healthcare products Regulatory Agency (MHRA) guidelines, where an “amber” result is defined as a white cell count (WCC) < 3.5 × 10^9^/L and ≥ 3.0 × 10^9^/L and/or ANC < 2.0 × 10^9^/L and ≥ 1.5 × 10^9^/L and a “red” result is defined as a WCC < 3.0 × 10^9^/L and/or ANC < 1.5 × 10^9^/L. These thresholds are lowered by 0.5 × 10^9^/L for patients with BEN. In our study, agranulocytosis was defined as an ANC < 0.5 × 10^9^/L. Where applicable, the time to agranulocytosis from clozapine initiation was examined. In the absence of agranulocytosis, red results were described as mild to moderate neutropenia in the study.

Other outcomes included all‐cause mortality during reduced haematological monitoring. In addition, we noted the proportion of patients who reverted to standard monitoring. Where applicable, we examined the number of patients who interrupted reduced monitoring but restarted prior to the end of follow‐up.

### 3.3 Statistical Analysis

Baseline demographics and clinical data were summarised using descriptive statistics. Frequencies and percentages were calculated for categorical data. Means and standard deviations were calculated for continuous data. Incidence rates of haematological events (red, amber results and agranulocytosis) were calculated as the number of cases divided by the number of person‐years. Person‐years were defined as the years contributed by each participant from the start date of reduced monitoring to the first haematological event, treatment discontinuation, or end of follow‐up. We used a Kaplan–Meier survival curve to estimate and graphically represent the time to all‐cause discontinuation from the date patients were changed from 4‐weekly to 12‐weekly monitoring intervals. Patients were followed up on from the date they were registered for reduced monitoring and were censored if they remained on reduced monitoring when lost to follow up or until 31 July 2024, whichever occurred sooner. Patients who were lost to follow up included transfers out of the Trust and death. Statistical analysis was performed using R Studio 2020 for Windows (RStudio PBC, Boston, USA; https://posit.co/products/open‐source/rstudio/).

## 4 Results

### 4.1 Patient Characteristics

In total, 1025 patients were prescribed clozapine and monitored at 3‐monthly intervals, which equated to a total of 3366 patient‐years and a median follow‐up time of 4 years (IQR 1) per participant. The mean age of the cohort was 47 years (SD 12); 69% were male, and the mean duration of clozapine was 10 years prior to extended monitoring (SD 5). See Table 1.

**TABLE 1:** Sociodemographic and clinical characteristics of patients receiving reduced monitoring.

Characteristics	Total (*N* = 1025)
Gender *n* (%)	
Male	704 (69)
Female	321 (31)
Age at reduced monitoring registration (years)	
Mean (SD)	47 (11)
Median (IQR)	48 (16)
Benign ethnic neutropenia monitoring *n* (%)	40 (4)
Diagnosis *n* (%)	
F20 Paranoid Schizophrenia	1025 (100)
Ethnicity *n* (%)	
White	490 (48)
Black	307 (30)
Asian	113 (11)
Mixed	33 (3)
Other	82 (8)
Duration of clozapine treatment	
Mean (years ± SD)	10 (5)
Median (IQR)	12 (6)
Previous haematological aberration *n* (%)	83 (8)

### 4.2 Haematological Characteristics

During the follow up period, nine of the 1025 patients (0.9%) recorded a red result and 34 patients (3.3%) recorded an amber result. There were no cases of agranulocytosis. The incidence rate of haematological events per 100 person‐years was 1.28. The incidence rate of red and amber results per 100 person‐years was 0.27 and 1.01, respectively. A summary of haematological characteristics can be found in Table 2.

**TABLE 2:** Haematological outcomes in reduced monitoring cohort.

Characteristics	Total (*N* = 1025) *n* (%)
Patients with at least one amber result	34 (3.3)^a^
Patients with at least one (isolated) red result (%)	9 (0.9)
Patients with agranulocytosis (%)	0 (0)

^a^ Two of these patients were registered as having BEN.

### 4.3 Continuation Rates

Overall, 847 patients (83%) remained on reduced frequency blood monitoring throughout the entire study. In total, 21 patients (2%) discontinued reduced frequency monitoring and 157 patients (15%) temporarily interrupted reduced monitoring but restarted before the end of the follow‐up period. Eleven patients (1.08%) were lost to follow‐up. (See Table 3).

**TABLE 3:** Continuation of clozapine during 3‐monthly monitoring.

Outcome at end of follow‐up	Total (*n* = 1025) *n* (%)
Continued reduced monitoring without reversion to monthly	847 (83)^a^, ^b^
Discontinued reduced monitoring	21 (2)^b^
Interrupted reduced monitoring	157 (15)

^a^ 11 patients lost to follow up.

^b^ 42 patients had died.

### 4.4 Time to Discontinuation of Reduced Monitoring

**FIGURE 1:**
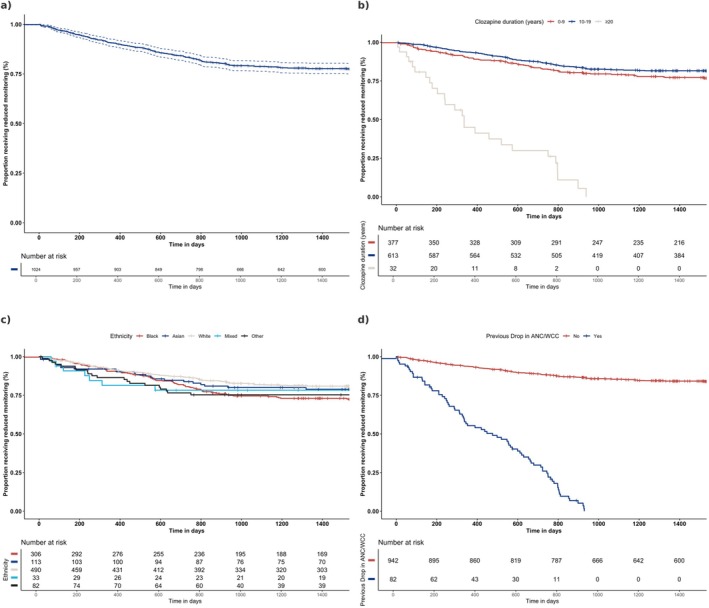
Kaplan–Meier curves of time to discontinuation of reduced monitoring. (a) Discontinuation for entire cohort. (b) Discontinuation stratified by ethnicity. (c) Discontinuation stratified by haematological aberration prior to reduced monitoring. (d) Discontinuation stratified by duration of clozapine treatment prior to reduced monitoring.

The Kaplan–Meier estimates for time until discontinuation of reduced monitoring are displayed in Figure 1.

### 4.5 Recorded Deaths

Of the total cohort, 42 patients (4%) died during the 3‐monthly monitoring period. Of these patients, none had amber or red results during the 3‐monthly monitoring period. Details are given in Table 4.

**TABLE 4:** Recorded causes of death during the 3‐monthly monitoring period.

Causes of death^a^	*N*
Bowel obstruction	1
Cancer	3
Cardiovascular diseases	9
Clozapine‐related^2^	1
Diabetes	2
Respiratory diseases	22
Seizure	1
Substance abuse	3
Unknown^b^	9

^a^ More than one cause may have been reported.

^b^ Not due to agranulocytosis.

## 5 Discussion

### 5.1 Findings

To the best of our knowledge, this is the largest study to evaluate outcomes of reduced frequency haematological monitoring in patients established on clozapine treatment. In this national sample of 1025 patients over 3366 patient‐years of exposure, there were no cases of agranulocytosis or of blood dyscrasias‐related deaths during 12‐weekly blood monitoring. Amongst our cohort, there were nine cases with neutrophil counts below 1.5 × 10^9^/L (a rate of 0.27 per 100 patient years), none of which progressed to agranulocytosis. Furthermore, most patients (98%) persisted with clozapine treatment and remained on reduced haematological monitoring. Considering the significant impediment haematological monitoring poses on clozapine utilisation rates, our findings have important clinical implications.

### 5.2 Comparison With Other Studies

Many previous studies have demonstrated the feasibility of reduced frequency haematological monitoring without compromising patient safety [10, 11, 12, 13, 14]. For example, a recent observational study in Canada evaluated the impact of reducing blood monitoring from 4 to 8‐week intervals in patients prescribed clozapine. The authors found no significant impact in the likelihood of detecting haematological aberrations in a cohort of 621 patients (OR = 0.83, 95% CI [0.58–1.41], *p* = 0.55). Consistent with these findings is the earlier work by Wadoo and colleagues who demonstrated a zero incidence of neutropenia and fewer admissions to psychiatric (1 [1.1%] vs. 3 [3.2%]) and medical (1 [1.1%] vs. 3 [3.2%]) facilities in patients receiving clozapine under a 12‐weekly monitoring regimen. Similarly, our previous analysis of an earlier sample of the current cohort found that median admission rates remained unchanged over 2 years before and after a reduction in haematological monitoring frequency (0, IQR = 0) [7]. It is noteworthy that, as with the current study, patients who received clozapine with reduced monitoring in the aforementioned studies were established on treatment for at least 12 months. Therefore, we cannot speculate on the effect of reduced monitoring on a more generalised population which includes patients prescribed clozapine for a shorter period. Notwithstanding this observation, the current evidence, alongside findings of the current study, supports calls to revise the mandatory haematological monitoring requirements for patients long‐established on clozapine treatment.

### 5.3 Clinical Implications

The expected rate of agranulocytosis in people taking clozapine for more than 1 year is 0.31–0.52 per 1000 person years [10], and so we might have expected to see at least one case (1.04–1.75) during our observation period. The fact that we saw no cases does not suggest that the risk of agranulocytosis in long term patients is less than previously thought, but it does suggest, albeit tentatively, that 3‐monthly monitoring is no less safe than the standard 1‐monthly monitoring.

Monitoring of neutrophil counts in clozapine patients has two purposes: to identify cases of agranulocytosis so that clozapine can be stopped, and to prevent cases occurring by identifying patients whose neutrophil count pattern suggests a possible impending agranulocytosis and stopping clozapine before agranulocytosis can develop. It is unlikely that 3‐monthly monitoring caused us to miss cases of agranulocytosis that might have been identified by monthly monitoring. We can be certain about this because CIA characteristically involves a rapid and catastrophic fall in neutrophil counts, which has obvious clinical consequences such as fever and severe infection [4]. The typical rapidity of the fall in neutrophils (normal to zero or near zero over 2 weeks) may also mean that 1‐monthly and 3‐monthly monitoring are similarly inadequate in their ability to detect developing agranulocytosis since, in both cases, the monitoring interval is substantially longer than the timescale of the condition. People taking clozapine would probably be better served by being asked to present promptly for blood testing whenever they develop a feverish illness. They might also carry an alert card or wear a warning bracelet, as is common with some longterm physical illnesses [15]. Comprehensive patient and carer education would clearly be necessary.

There is a critical need to reduce the barriers for the use of clozapine in treatment‐resistant psychosis. Considering existing research showing that haematological monitoring is a prominent barrier to increased clozapine use, policymakers should prioritise the relaxation of haematological monitoring frequency to improve patient care, from both a clinical and economic perspective [16, 17].

### 5.4 Strengths and Limitations

A particular strength of this study is the large sample size and long follow‐up time (a median [IQR] of 4 [1] years) that has the facility to capture late occurrences of CIA. Another strength of this study is the diversity of patients examined, with 30% of patients being of Black ethnicity and the range of clozapine treatment durations prior to reduced monitoring being up to 30 years. Limitations include the observational and non‐comparative study design and descriptive nature of statistical analyses that limit the conclusions that can be made. Nonetheless, when considered alongside existing evidence, our study strongly suggests that reduced haematological monitoring frequency is safe. Another limitation of our study is the highly selective nature of patients who received reduced haematological monitoring, restricting the generalisability of our study findings. Our study is also perhaps limited by the absence of a control group of people undergoing monthly monitoring. However, with no cases of agranulocytosis in our cohort and no deaths due to blood dyscrasia, comparison with another group would add little to our conclusions. While there were no CIA‐related deaths in our study, an equally important consideration is whether the other causes of death could have been prevented with more regular monitoring and clinician contact. This was beyond the scope of the present study and requires future investigation.

### 5.5 Incidental Findings

There were a striking number of deaths from respiratory disease (22) in this study. This is not the first study to suggest that clozapine is associated with pneumonia and other serious lung diseases. A recent naturalistic follow‐up study found that pneumonia occurred in 29.5% of people taking clozapine for a median of 12.7 years [18]. A substantially increased risk of lung infection is evident from the start of clozapine treatment [19]. Pneumonia is very clearly a much more important contributor to clozapine‐related mortality than is the risk of agranulocytosis.

## 6 Conclusion

This is the largest study of reduced frequency clozapine monitoring. In over 1000 patients receiving clozapine over an average of 4 years under reduced frequency monitoring, there were no deaths due to agranulocytosis. Our findings do not so much support a change from 4‐weekly to 12‐weekly monitoring but instead suggest the abandonment of haematological monitoring in long‐term patients and the adoption of a patient education initiative aimed at ensuring that patients present to medical services as soon as any feverish illness develops. Such a change would likely go some way to improving persistence with clozapine treatment. Measures aimed at reducing the risk of pneumonia (e.g., pneumococcal vaccination) should be introduced in people taking clozapine.

## Author Contributions

Concept and design: David Taylor, Ebenezer Oloyede, Marinka Helthuis, Siobhan Gee. Drafting of manuscript: David Taylor, Ebenezer Oloyede, Siobhan Gee. Approval of manuscript: David Taylor, Ebenezer Oloyede, Marinka Helthuis, Siobhan Gee. Data provision: Marinka Helthuis. Data analysis: David Taylor, Ebenezer Oloyede.

## Conflicts of Interest

D.T. has received personal fees from H Lundbeck and Janssen unrelated to this study. Remaining authors declare no conflicts of interest.

## Peer Review

The peer review history for this article is available at https://www.webofscience.com/api/gateway/wos/peer‐review/10.1111/acps.13818.

## Supporting information


**Data S1.** Supporting Information.

## Data Availability

Data sharing is not applicable to this article as no new data were created or analyzed in this study.

## References

[1] J. Kane , G. Honigfeld , J. Singer , and H. Meltzer , “Clozapine for the Treatment‐Resistant Schizophrenic. A Double‐Blind Comparison With Chlorpromazine,” Archives of General Psychiatry 45, no. 9 (1988): 789–796, 10.1001/archpsyc.1988.01800330013001. 3046553

[2] E. Oloyede , G. Blackman , E. Whiskey , et al., “Clozapine haematological monitoring for neutropenia: a global perspective,” Epidemiology and Psychiatric Sciences 31 (2022): e83, 10.1017/S204579602200066X. 36426600 PMC9714212

[3] P. F. J. Schulte , S. R. T. Veerman , B. Bakker , J. Bogers , A. Jongkind , and D. Cohen , “Risk of Clozapine‐Associated Agranulocytosis and Mandatory White Blood Cell Monitoring: Can the Regulations Be Relaxed?,” Schizophrenia Research 268 (2024): 74–81, 10.1016/j.schres.2023.09.024. 37770377

[4] D. Taylor , K. Vallianatou , E. Whiskey , O. Dzahini , and J. MacCabe , “Distinctive Pattern of Neutrophil Count Change in Clozapine‐Associated, Life‐Threatening Agranulocytosis,” NPJ Schizophrenia 8, no. 1 (2022): 21, 10.1038/s41537-022-00232-0. PMC8920060 35288577

[5] J. M. J. Alvir , J. A. Lieberman , A. Z. Safferman , J. L. Schwimmer , and J. A. Schaaf , “Clozapine‐Induced Agranulocytosis—Incidence and Risk Factors in the United States,” New England Journal of Medicine 329, no. 3 (1993): 162–167. 8515788 10.1056/NEJM199307153290303

[6] D. Siskind , W. G. Honer , S. Clark , et al., “Consensus Statement on the Use of Clozapine During the COVID‐19 Pandemic,” Journal of Psychiatry & Neuroscience 45, no. 3 (2020): 222–223. 32297722 10.1503/jpn.200061 PMC7828973

[7] E. Oloyede , O. Dzahini , Z. Abolou , et al., “Clinical Impact of Reducing the Frequency of Clozapine Monitoring: Controlled Mirror‐Image Cohort Study,” British Journal of Psychiatry 223, no. 2 (2023): 1–7, 10.1192/bjp.2023.44. PMC10391318 37092691

[8] J. P. Vandenbroucke , E. von Elm , D. G. Altman , et al., “Strengthening the Reporting of Observational Studies in Epidemiology (STROBE): Explanation and Elaboration,” International Journal of Surgery 12, no. 12 (2014): 1500–1524, 10.1016/j.ijsu.2014.07.014. 25046751

[9] BVLD , Zaponex 100 mg Tablets, accessed July 17, 2020, https://www.medicines.org.uk/emc/product/7715/smpc.

[10] P. F. Schulte , “Risk of Clozapine‐Associated Agranulocytosis and Mandatory White Blood Cell Monitoring,” Annals of Pharmacotherapy 40, no. 4 (2006): 683–688, 10.1345/aph.1G396. 16595571

[11] M. Hata , M. Fujimoto , K. Kanai , et al., “No Adverse Events Were Observed in Clozapine‐Treated Patients on Extended Hematologic Monitoring Intervals During the Coronavirus Pandemic in Four Psychiatric Centers in Japan,” Neuropsychopharmacology Reports 41 (2021): 179–184. 33606356 10.1002/npr2.12166 PMC8013689

[12] K. Matsui , M. Ishibashi , M. Kawano , et al., “Clozapine‐Induced Agranulocytosis in Japan: Changes in Leukocyte/Neutrophil Counts Before and After Discontinuation of Clozapine,” Human Psychopharmacology 35, no. 4 (2020): e2739, 10.1002/hup.2739. 32420645

[13] O. Ingimarsson , J. H. MacCabe , M. Haraldsson , H. Jónsdóttir , and E. Sigurdsson , “Clozapine Treatment and Discontinuation in Iceland: A National Longitudinal Study Using Electronic Patient Records,” Nordic Journal of Psychiatry 70, no. 6 (2016): 450–455, 10.3109/08039488.2016.1155234. 27049594

[14] H. Thai , N. Preobrazenski , T. Hsieh , C. Robertson , and O. Owoeye , “Evaluating Reduced Blood Monitoring Frequency and the Detection of Hematological Abnormalities in Clozapine‐Treated Patients With Schizophrenia: A Chart Review Study From the COVID‐19 Pandemic,” Schizophrenia Bulletin 51, no. 2 (2024): sbae113, 10.1093/schbul/sbae113. PMC11908850 38984851

[15] G. Mattison , O. Canfell , D. Forrester , et al., “The Influence of Wearables on Health Care Outcomes in Chronic Disease: Systematic Review,” Journal of Medical Internet Research 24, no. 7 (2022): e36690, 10.2196/36690. 35776492 PMC9288104

[16] S. Parkes , B. Mantell , E. Oloyede , and G. Blackman , “Patients' Experiences of Clozapine for Treatment‐Resistant Schizophrenia: A Systematic Review,” Schizophrenia Bulletin Open 3 (2022): sgac042, 10.1093/schizbullopen/sgac042. 39144802 PMC11205966

[17] F. R. Girardin , A. Poncet , M. Blondon , et al., “Monitoring White Blood Cell Count in Adult Patients With Schizophrenia Who Are Taking Clozapine: A Cost‐Effectiveness Analysis,” Lancet Psychiatry 1, no. 1 (2014): 55–62, 10.1016/s2215-0366(14)70245-7. 26360402

[18] J. J. Partanen , P. Häppölä , A. Kämpe , et al., “High Burden of Ileus and Pneumonia in Clozapine‐Treated Individuals With Schizophrenia: A Finnish 25‐Year Follow‐Up Register Study,” American Journal of Psychiatry 181, no. 10 (2024): 879–892, 10.1176/appi.ajp.20230744. 39262212

[19] S. Mace , O. Dzahini , V. Cornelius , H. Langerman , E. Oloyede , and D. Taylor , “Incident Infection During the First Year of Treatment ‐ A Comparison of Clozapine and Paliperidone Palmitate Long‐Acting Injection,” Journal of Psychopharmacology 36, no. 2 (2022): 2698811211058973, 10.1177/02698811211058973. 34991402

